# Catechol-O-methyltransferase gene haplotypes in Mexican and Spanish patients with fibromyalgia

**DOI:** 10.1186/ar2316

**Published:** 2007-10-26

**Authors:** Gilberto Vargas-Alarcón, José-Manuel Fragoso, David Cruz-Robles, Angélica Vargas, Alfonso Vargas, José-Ignacio Lao-Villadóniga, Ferrán García-Fructuoso, Manuel Ramos-Kuri, Fernando Hernández, Rashidi Springall, Rafael Bojalil, Maite Vallejo, Manuel Martínez-Lavín

**Affiliations:** 1 National Institute of Cardiology, Juan Badiano 1, Mexico City 14080, Mexico; 2 Dr. Echevarne Laboratory, Provenza 312, Barcelona E08037, Spain; 3 CIMA Clinic, Manuel Girona 33, Barcelona E08034, Spain; 4 Panamerican University, Donatelo 59, Mexico City 03920, Mexico

## Abstract

Autonomic dysfunction is frequent in patients with fibromyalgia (FM). Heart rate variability analyses have demonstrated signs of ongoing sympathetic hyperactivity. Catecholamines are sympathetic neurotransmitters. Catechol-O-methyltransferase (COMT), an enzyme, is the major catecholamine-clearing pathway. There are several single-nucleotide polymorphisms (SNPs) in the COMT gene associated with the different catecholamine-clearing abilities of the COMT enzyme. These SNPs are in linkage disequilibrium and segregate as 'haplotypes'. Healthy females with a particular COMT gene haplotype (ACCG) producing a defective enzyme are more sensitive to painful stimuli. The objective of our study was to define whether women with FM, from two different countries (Mexico and Spain), have the COMT gene haplotypes that have been previously associated with greater sensitivity to pain. All the individuals in the study were female. Fifty-seven Mexican patients and 78 Spanish patients were compared with their respective healthy control groups. All participants filled out the Fibromyalgia Impact Questionnaire (FIQ). Six COMT SNPs (rs2097903, rs6269, rs4633, rs4818, rs4680, and rs165599) were genotyped from peripheral blood DNA. In Spanish patients, there was a significant association between three SNPs (rs6269, rs4818, and rs4680) and the presence of FM when compared with healthy controls. Moreover, in Spanish patients with the 'high pain sensitivity' haplotype (ACCG), the disease, as assessed by the FIQ, was more severe. By contrast, Mexican patients displayed only a weak association between rs6269 and rs165599, and some FIQ subscales. In our group of Spanish patients, there was an association between FM and the COMT haplotype previously associated with high pain sensitivity. This association was not observed in Mexican patients. Studies with a larger sample size are needed in order to verify or amend these preliminary results.

## Introduction

Fibromyalgia (FM) is a frequent illness. Epidemiological studies from different parts of the world have shown a prevalence rate of 2% to 3% in the general population. Approximately 90% of affected individuals are female [1]. The severity of the illness diminishes the quality of life of afflicted persons [2] and imposes a heavy economic burden on society [3]. Therefore, FM can be considered a major health problem among contemporary women.

Different groups of investigators have shown that autonomic dysfunction is frequent in patients with FM. Heart rate variability analyses have demonstrated signs of ongoing sympathetic hyperactivity. This hyperactivity is accompanied by a blunted sympathetic response to different stressors. It has been proposed that this autonomic dysfunction explains the multifaceted complaints of FM [4]. The defining characteristics of FM (namely, widespread pain and allodynia) could be explained by a pathogenesis known as 'sympathetically maintained pain' [5].

Naturally occurring sympathetic neurotransmitters are catecholamines known as norepinephrine, epinephrine, and dopamine. All three substances act within the central nervous system. Norepinephrine acts also in peripheral postganglionic nerve endings and exerts local effects in close proximity to the area where it is released, whereas epinephrine is the circulating hormone of the adrenal medulla and influences processes throughout the body.

The major systemic transformation of catecholamines is catalyzed by the catechol-O-methyltransferase (COMT) enzyme. The COMT gene is located in region 22q11.1 to 22q11.2 of chromosome 22. There are different single-nucleotide polymorphisms (SNPs) in the COMT gene which induce important functional alterations of the enzyme. The best-studied SNP (rs4680) occurs in codon 158 with valine-to-methionine transition (Val-158-Met). The Val-158-Val genotype gives rise to an effective enzyme, whereas the Met-158-Met genotype produces a defective enzyme, which is incapable of effectively clearing catecholamines from the system [6].

Zubieta and colleagues [7] demonstrated that healthy individuals with the COMT Val-158-Val genotype are pain-resistant. The opposite occurs in people with the Met-158-Met genotype. Gursoy and colleagues [8], of Turkey, reported an association between FM and the Val-158-Met COMT polymorphism. García-Fructuoso and colleagues [9], of Spain, described that FM patients with the Met-158-Met genotype have a more severe form of the disease when compared with affected individuals with the Val-158-Val genotype.

More meticulous genetic studies demonstrated that COMT functional properties are only marginally dependent on the Val-158-Met transition. There are other SNPs in the COMT gene (rs6269, rs4633, and rs4818) in linkage disequilibrium (LD) with the Val-158-Met variation. By combining these SNPs, Diatchenko and colleagues [10] were able to identify frequent COMT gene haplotypes strongly associated with sensitivity to experimental pain and induction of a more defective COMT enzyme. Healthy females with the 'high pain sensitivity haplotype' have a COMT enzyme 11 times less efficient than persons with the 'low pain sensitivity haplotype'. Diatchenko and colleagues [10] also demonstrated that COMT inhibition in rats results in a dramatic increase in sensitivity to pain. Low COMT activity may increase pain sensitivity by activating beta (2/3)-adrenergic receptors. The objective of our investigation was to define whether FM patients from two different countries (Mexico and Spain) have COMT alleles associated with increased susceptibility to pain in healthy individuals.

## Materials and methods

### Patients

All the subjects studied were women. The criterion for inclusion was to be diagnosed with FM as per the guidelines of the American College of Rheumatology and to be free of concurrent rheumatic disease. These patients were sourced from different private rheumatology practices in Mexico and Spain. Mexican controls were women who considered themselves to be healthy and who denied having chronic pain. Twenty-seven of them were paramedical personnel and 6 were relatives of paramedical personnel but were not related among themselves. Spanish controls were blood donors who denied having chronic pain. Patients and controls were matched by gender and age. Informed consent was obtained from all participants, and the Human Research Committee of the National Institute of Cardiology of Mexico approved the study.

Both patients and controls filled out a validated Spanish translation of the Fibromyalgia Impact Questionnaire (FIQ) [11]. This is an instrument designed to numerically define the overall impact of FM across many dimensions (for example, function, pain level, fatigue, sleep disturbance, and psychological distress) [12].

### Genotyping

Genomic DNA from whole blood containing EDTA (ethylenediaminetetraacetic acid) was extracted by standard techniques [13]. Six COMT SNPs (rs2097903, rs6269, rs4633, rs4818, rs4680, and rs165599) were genotyped using 5' exonuclease TaqMan assays on an ABI Prism 7000 Sequence Detection System (Applied Biosystems, Foster City, CA, USA) according to the manufacturer's instructions. The National Center for Biotechnology Information (Bethesda, MD, USA) SNP database was used to assign SNP numbers.

### Statistical analysis

The χ^2 ^test was used to evaluate the Hardy-Weinberg equilibrium for each polymorphism. Statistical analysis was carried out with Stata 8.0 for Windows software (College Station, TX, USA). During the exploratory analysis, our numerical data showed a non-Gaussian distribution (Shapiro-Wilk test, *p *> 0.05). Therefore, Kruskal-Wallis tests were used to compare these variables. Data are presented as median and 25th and 75th percentiles. Categorical variables were analyzed with χ^2 ^or Fisher tests as required and presented as absolute frequencies and proportions. Statistical significance was set at an alpha level of less than or equal to 0.05. The total FIQ score was correlated with genotypes and haplotypes. Mexican and Spanish groups were examined separately. Pairwise linkage disequilibrium (LD, D') estimations between polymorphisms and haplotype reconstruction were performed with Haploview version 3:32 (Broad Institute of Massachusetts Institute of Technology and Harvard University, Cambridge, MA, USA).

## Results

Table 1 shows the demographic data of the populations studied. The two groups of patients had similar age distributions and FIQ scores. However, Mexican controls had higher FIQ scores when compared with Spanish controls (*p *< 0.05).

**Table 1:** Demographic data of the studied populations

	Mexican		Spanish	
	Patients	Controls	Patients	Controls

Number	57	33	78	80
Mean age in years (SD)	45 (12)	45 (12)	47 (7)	44 (9)
FIQ score (SD)	70 (14)	5.9 (6.3)^a^	68 (11)	1.6 (2.5)^a^

All tested individuals were women. Mexican controls had a higher Fibromyalgia Impact Questionnaire (FIQ) score when compared with Spanish controls (*p *< 0.05) ^a^. SD, standard deviation.

Genotype distribution of all COMT SNPs studied in both populations is shown in Table 2. The observed and expected frequencies of the different SNPs in both populations were in Hardy-Weinberg equilibrium. Statistically significant associations were found between three SNPs (rs6269, rs4818, and rs4680) and the development of FM in Spaniards. In Mexicans, the distribution of the six SNPs did not differ between patients and controls.

**Table 2:** Genotype distribution of the six catechol-O-methyltransferase single-nucleotide polymorphisms in patients and healthy controls from Mexico and Spain

	Spanish			Mexican		
Genotype	Patients	Controls	*P *value	Patients	Controls	*P *value
	n (%)	n (%)		n (%)	n (%)	
rs6269						
AA	21 (27)	14 (18)	0.015^a^	30 (53)	17 (52)	NS
AG	42 (54)	34 (42)		21 (37)	13 (39)	
GG	15 (19)	32 (40)		6 (10)	3 (9)	
rs4633						
CC	28 (36)	19 (24)	NS^a^	21 (37)	17 (52)	NS
CT	36 (46)	37 (46)		28 (49)	13 (39)	
TT	14 (18)	24 (30)		8 (14)	3 (9)	
rs4818						
CC	19 (24)	13 (16)	0.001	30 (53)	15 (46)	NS
CG	47 (60)	33 (41)		21 (36)	12 (36)	
GG	12 (16)	34 (43)		6 (11)	6 (18)	
rs4680						
AA	9 (12)	22 (27)	0.023^a^	2 (4)	4 (12)	NS
AG	40 (51)	39 (49)		32 (56)	14 (42)	
GG	29 (37)	19 (24)		23 (40)	15 (46)	
rs20907						
AA	31 (40)	23 (29)	NS^a^	26 (46)	15 (45.5)	NS
AG	34 (43)	40 (50)		24 (42)	15 (45.5)	
GG	13 (17)	17 (21)		7 (12)	3 (9)	
rs16559						
AA	36 (46)	33 (41)	NS^a^	12 (21)	10 (30)	NS^a^
AG	30 (39)	35 (44)		32 (56)	15 (46)	
GG	12 (15)	12 (15)		13 (23)	8 (24)	

^a^χ^2 ^test; NS, not significant.

Table 3 depicts the correlation between the SNPs and total FIQ score. Again, there was a strong correlation between high FIQ score and four SNP genotypes (rs6269AA, rs4633CC, rs4818CC, and rs4680GG) in Spaniards. No correlation was evident in Mexicans.

**Table 3:** Comparison of single-nucleotide polymorphisms and Fibromyalgia Impact Questionnaire scores in patients from Mexico and Spain

	Spaniards				Mexicans			
Genotypes	25th percentile	Median	75th percentile	*P *value	25th percentile	Median	75th percentile	*P *value
rs6269								
AA	75.1	78.6	82.1		57.7	67.53	80.5	
AG	60.6	67.1	70.1	0.0001	68.7	77.5	80.67	0.193
GG	51.1	56.3	56.7		41.01	66.77	79.17	
rs4633								
CC	73.9	78.7	86.1		61.4	71.25	78.17	
CT	59.8	62.6	68.5	0.0001	60.7	71.29	82.7	0.785
TT	51.1	56.2	56.7		57.7	66.21	85.47	
rs4818								
CC	75.1	78.1	82.4		57.7	64.20	80.5	
GC	60.2	65.3	70.2	0.0001	68.74	77.5	81.58	0.054
GG	51.7	56.2	61.7		60.43	66.33	73.17	
rs4680								
AA	50.62	52.3	56.1		57.7	72.97	88.24	
AG	59.4	62.1	68.3	0.0001	62.7	74.35	83.60	0.284
GG	72.8	78.6	85		59.9	68.74	76.46	

Only single-nucleotide polymorphisms with significant associations in Spaniards are shown. In Mexicans, none was associated with the Fibromyalgia Impact Questionnaire.

FIQ contains several visual analogue scales. In Spaniards, there was an association between the above-mentioned SNPs and the visual analogue scale scores for pain fatigue, sleep disturbance, and morning stiffness. In Mexicans, a significant correlation was found between rs6269 and pain and fatigue as well as between rs165599 and disability and morning stiffness.

LD analyses between the six SNP markers showed that four of them – rs6269, rs4633, rs4818, and rs4680 (located in the central region of the COMT gene) – had strong LDin both populations. The strongest associations were found between SNPs rs6269 with rs4680, rs6269 with rs4818, and rs4633 with rs4680 (D' = 0.986, *R*^2 ^= 0.878). In light of this finding, we analyzed the most frequent haplotypes in patients and healthy controls to determine whether some of these haplotypes could be associated with the risk of developing FM among the populations in the study. There were four frequent haplotypes in Spaniards (ACCG, ATCA, GCGG, and GTGA). Haplotype distribution was different in Spanish patients versus controls (*p *= 0.006). Patients had higher ACCG and ATCA frequencies and a lower GTGA frequency. Mexicans had three frequent haplotypes (ACCG, ATCA, and GCGG) that were distributed similarly among patients and controls (Table 4). In Spanish patients, the ACCG haplotype was strongly associated with a high-percentile FIQ score (*p *= 0.0001) (Table 5).

**Table 4:** Haplotype frequencies in patients and healthy controls from Mexico and Spain

	Spanish		Mexican	
	Patients	Controls	Patients	Controls

	(*n *= 78)	(*n *= 80)	(*n *= 57)	(*n *= 33)

Haplotypes				
ACCG	46 (0.295)	31 (0.194)	33 (0.289)	19 (0.287)
ATCA	34 (0.218)	26 (0.162)	31 (0.272)	18 (0.272)
GCGG	41 (0.263)	42 (0.262)	23 (0.201)	16 (0.242)
GTGA	24 (0.154)	52 (0.325)	0	0
Other	11 (0.070)	9 (0.056)	27 (0.236)	13 (0.197)
Total of haplotypes	156	160	114	66
*P *values	0.006		NS	

Each frequency is calculated by dividing the occurrence of a given haplotype by the total number of haplotypes in that particular group (haplotype total = number of individuals × 2). NS, not significant.

**Table 5:** Comparison of total Fibromyalgia Impact Questionnaire score and haplotypes using Kruskal-Wallis test

	Spaniards				Mexicans			
	25th percentile	Median	75th percentile	*P *value	25th percentile	Median	75th percentile	*P *value
Haplotypes								
ACCG	72.78	78.09	82.42	0.0001	53.54	70.05	77	NS
ATCA	60.21	62.87	68.46		59.6	68.18	84.5	
GCGG	60.4	67.16	69.56		60.43	74.36	79.17	
GTGA	51.11	56.1	56.41		-	-	-	

NS, not significant.

## Discussion

FM strongly aggregates in families and coaggregates with mood disorders [14]. Nevertheless, previous attempts to show a specific genetic defect in FM yielded weak and/or divergent results. In a multicase family study, Yunus and colleagues [15] found a feeble association with the human leukocyte antigen (HLA) region. The reported relationship to the serotonin transporter gene (*5-HTTLPR*) [16] is probably linked to comorbid depression rather than to FM itself [17]. Studies on the dopamine D4 exon III repeat polymorphism yield inconsistent results [18].

In view of the sympathetic dysfunction demonstrated in FM, the COMT gene became an attractive genetic target. Exploratory studies by Gursoy and colleagues [8] and García-Fructuoso and colleagues [9] uncovered an association between the Val-158-Met (rs4680) COMT transition and FM. In contrast, Hagen and colleagues [19], using mail questionnaires, found no association between Val-158-Met transition and chronic musculoskeletal pain. Since there was no direct contact with patients, the percentage of people with musculoskeletal pain actually had FM was not established. Another report by the same group using the same method described an association between Val-158-Met polymorphism and headache [20].

In the study of Diatchenko and colleagues [10] of healthy female volunteers, SNP rs4818 accounted for 7% of pain sensitivity, SNP rs6269 for 6%, and SNP rs4680 (Val-158-Met) showed only a marginal relationship with pain sensitivity. Our results in the Spanish population are in line with these considerations. Such SNPs were associated not only with the diagnosis of FM, but also with the severity of the illness as assessed by FIQ scores. Additionally, we found SNP rs4633 to be associated with FM in Spaniards. This SNP, which represents a synonymous variation (does not produce a change in amino acid composition), was not found to be associated with pain sensitivity in the study of Diatchenko and colleagues [10].

The differences observed between Spanish and Mexican populations were an unexpected result of our study. We view two possible explanations for such a discrepancy.

1. Population differences. The Mexican population has been shown to be one of the most vigorous genetic admixtures, with genes of Caucasian, Amerindian, and African origin. A parental group represented by Spaniards has a more homogeneous background. Vargas-Alarcón and colleagues [21] analyzed the HLA allele distribution in 69 populations around the world, including Mexicans and Spaniards. Genetic distances with corresponding analyses showed important differences between these two groups [21]. Previously, it has been recognized that, in the presence of COMT alleles, there will be ethnic variations. Such variations were found not only among the general population, but also in association with disease [6].

2. Sampling error and other stratifications. Control groups were different. Mexicans were paramedical personnel and other women who considered themselves to be healthy. Paramedical personnel are not the most representative of all people without FM in the population from which the FM cases were obtained. Mexican controls had higher FIQ scores than their blood-donor Spanish counterparts. We consider that this bias is unlikely to explain our diverging results. Even after the Mexican controls with the highest FIQ score were excluded from our calculations, differences persisted. Furthermore, although Mexican and Spanish patients had similar FIQ scores, they displayed a significantly different distribution in COMT SNP rs6269, rs16559, and rs4818.

Several reports suggest that the collective grouping of SNPs in haplotypes has a stronger association with the assessed phenotype. In our study, COMT haplotype analysis showed a strong association between ACCG and ATCA and the risk of developing FM in Spanish individuals. Additionally, the ACCG haplotype was associated with a high FIQ score in Spanish patients. Previously, this haplotype has been designated as 'high pain sensitivity' due to the fact that healthy women with this particular genetic make-up are much more sensitive to painful stimuli. Our results suggest that an ACCG haplotype could be an important susceptibility marker for FM in the Spanish population.

## Conclusion

Our results show an association between FM and the COMT pain sensitivity SNPs in Spaniards. By contrast, the Mexican population displayed only a weak association between the two SNPs and isolated FM symptoms. This phenomenon suggests that there are population variations in susceptibility to developing FM which are related to the COMT gene polymorphism. Studies with a larger sample size are needed in order to verify or amend these preliminary results.

## Abbreviations

COMT = catechol-O-methyltransferase; FIQ = Fibromyalgia Impact Questionnaire; FM = fibromyalgia; HLA = human leukocyte antigen; LD = linkage disequilibrium; SNP = single-nucleotide polymorphism.

## Competing interests

The authors declare that they have no competing interests.

## Authors' contributions

GVA, JMF, DCR, MRK, and FH carried out the molecular genetic studies. FGF and JILV recruited and studied the Spanish population and purified DNA samples. RB and RS purified DNA samples from Mexico. AV and AV performed the clinical studies on Mexican participants. MV carried out the statistical analyses. MML conceived the study, participated in its design and coordination, and helped to draft the manuscript. All authors read and approved the final manuscript.
